# Application of long-read sequencing to the detection of structural variants in human cancer genomes

**DOI:** 10.1016/j.csbj.2021.07.030

**Published:** 2021-07-28

**Authors:** Yoshitaka Sakamoto, Suzuko Zaha, Yutaka Suzuki, Masahide Seki, Ayako Suzuki

**Affiliations:** Department of Computational Biology and Medical Sciences, Graduate School of Frontier Sciences, The University of Tokyo, 5-1-5 Kashiwanoha, Kashiwa, Chiba 277-8561, Japan

**Keywords:** Long-read sequencing, Structural variant, Cancer genome

## Abstract

In recent years, the so-called long-read sequencing technology has had a substantial impact on various aspects of genome sciences. Here, we introduce recent studies of cancerous structural variants (SVs) using long-read sequencing technologies, namely Pacific Biosciences (PacBio) sequencers, Oxford Nanopore Technologies (ONT) sequencers, and linked-read methods. By taking advantage of long-read lengths, these technologies have enabled the precise detection of SVs, including long insertions by transposable elements, such as LINE-1. In addition to SV detection, the epigenome status (including DNA methylation and haplotype information) surrounding SV loci has also been unveiled by long-read sequencing technologies, to identify the effects of SVs. Among the various research fields in which long-read sequencing has been applied, cancer genomics has shown the most remarkable advances. In fact, many studies are beginning to shed light on the detection of SVs and the elucidation of their complex structures in various types of cancer. In the particular case of cancers, we summarize the technical limitations of the application of this technology to the analysis of clinical samples. We will introduce recent achievements from this viewpoint. However, a similar approach will be started for other applications in the near future. Therefore, by complementing the current short-read sequencing analysis, long-read sequencing should reveal the complex nature of human genomes in their healthy and disease states, which will open a new opportunity for a better understanding of disease development and for a novel strategy for drug development.

## Graphical abstract

**Figure:**
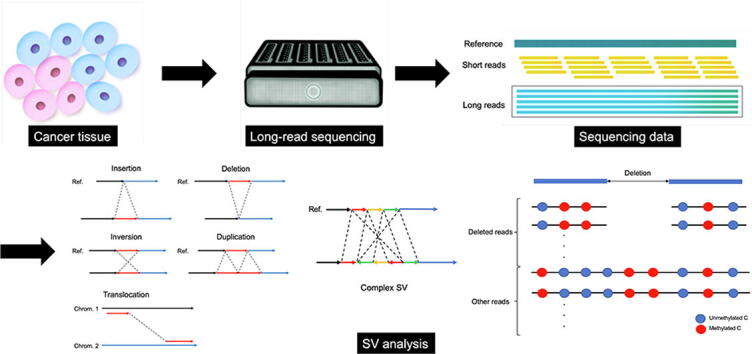

## 1 Introduction

**Fig. 1:**
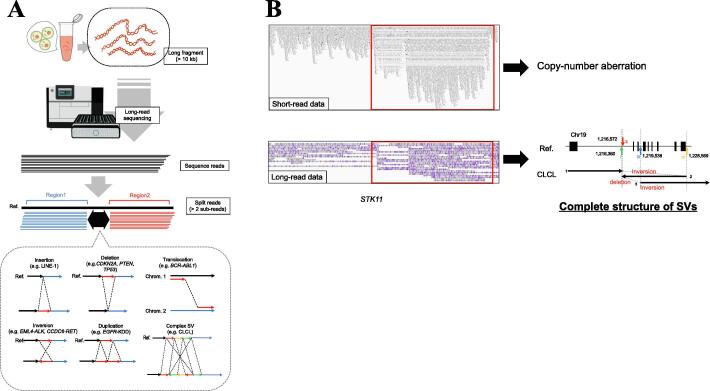
Examples of structural variants (SVs). (A) The workflow of detection of SVs. Long DNA fragment (>10 kb) is extracted. Then, long-read sequencing is conducted. Sequence reads that support SVs are mapped to multiple regions of the reference genome by splitting a read into multiple sub-reads. Representative SVs, such as insertions, deletions, inversions, duplications, translocations, and complex SVs are shown. The complex SVs indicate the combination of inversions and duplications. The red and green arrows indicate inverted duplications, and the yellow arrow indicates an inversion. Cancer-related biological events involving each SV are introduced. For example, LINE-1 insertion [12], deletions of tumor suppressor genes such as *CDKN2A*, *PTEN*, and *TP53* genes [95], *BCR-ABL1* fusion gene by translocation [96], *EML4-ALK*[82] and *CCDC6-RET* fusion gene [97] by inversion, *EGFR* kinase domain duplication (KDD), and CLCL as complex SV [61]. (B) An example of CLCL that indicates a complex SV with the combination of inversion, deletion, and duplication. The CLCL was identified in the *STK11* gene which is a tumor suppressor gene and a marker of immune checkpoint inhibition. IGV view of short-read sequencing data (upper left) and long-read sequencing data (lower left). Short-read sequencing data represented copy-number aberrations in the region. Long-read sequencing data can reconstruct the structure of the copy-number aberration as follows (lower right). 1) Inversion from junction II (red arrow) to junction IV (yellow allow); 2) deletion from junction I (red arrow) to junction III (blue arrow); and 3) Inversion following junction III. (For interpretation of the references to colour in this figure legend, the reader is referred to the web version of this article.)

Long-read DNA sequencers have contributed significantly to our knowledge of structural variants (SVs) in chromosomes. SVs include large insertions and deletions (indels), inversions, duplications, translocations, and complex combinations of these mutations, and are at least 50 bp in length (Fig. 1A) [1]. SVs exist in all genomes as a form of genetic variation, and researchers have been trying to construct a catalog of SVs in the human genome using long-read and short-read sequencing technologies [2], [3]. SVs sometimes affect human diseases, such as Mendelian disorders, autism, and cancer [4].

Cancer genomics is one of the representative fields in which the long-read sequencing technologies have already achieved significant results. In cancers in particular, SVs occurring somatically in unstable cancerous genomes may affect the functions of several oncogenes and tumor suppressor genes. In lung cancers, for example, fusion genes, such as *RET*, *ALK*, and *ROS1*, which contribute to tumorigenesis as driver mutations [5], are examples of cancer-promoting SVs. Similarly, *ERBB2* (*HER2*) amplification plays a pivotal role in breast cancers [6]. Cancer genome mutations, including these SVs, have mostly been analyzed using short-read sequencing. The Pan-Cancer Analysis of Whole Genomes (PCAWG) project identified and characterized different types of SVs in cancer genomes [7], [8], [9], [10], [11], [12], [13]. Using the PCAWG data, three types of novel complex rearrangements, namely pyrgo, rigma, and tyfonas, were identified [14]. Those rearrangements indicate complex copy-number variations, which include duplications, deletions, and inversions based on junction analysis. Pyrgo is constructed by multiple duplications with a low junction copy number. Rigma is constructed by multiple deletions. Tyfonas is constructed by multiple duplications and inversions with a high junction copy number. However, it is difficult to precisely detect SVs and their complicated structures using short-read sequencing, simply because the read length does not exceed the size of the SVs.

To address this concern, long-read sequencing technologies have been rapidly evolving, as follows.

1) Pacific Biosciences (PacBio) developed single-molecule real-time (SMRT) sequencing using a DNA polymerase in a zero-mode waveguide [15]. In 2019, PacBio also developed circular consensus sequencing (CCS), which was able to achieve high base accuracy from a noisy original long-read [16]. CCS generates a consensus read from a single template via the circulation of double-stranded DNA and attached DNA polymerase. Using this approach, a base accuracy of over 99% can be achieved in sequences of about 13 kb in length. Of note, around 10 µg of DNA is required as the input in this process, which sometimes imposes a serious burden for a series of targets for which only a limited amount of DNA is available. These targets include small cancers or cancers at an early stage.

2) MinION and PromethION, which are Nanopore-type sequencers, have been developed by Oxford Nanopore Technologies (ONT). Nanopore-type sequencers recognize bases according to the differences produced in electronic signals when the bases pass through protein nanopores parallelized in a flowcell. MinION is a Nanopore-type portable sequencer that was originally commercialized with a read length of around 10 kb and the throughput of a single flowcell of around 5 Gb [17], [18]. The PromethION platform has increased the number of pores per flowcell and enabled the running of multiple flowcells, with up to 48 flowcells operating simultaneously. The sequencing yields of PromethION are substantial, at more than 100 Gb per flowcell, giving a maximum capacity 48 times higher than this and producing read lengths of more than 50 kb at N50 and up to around 1 Mb [19], [20]. Generally, it is supposed that an ONT sequencer can produce longer reads than a PacBio sequencer. In addition, the input DNA required is, to some extent, lower than that of the PacBio sequencer, although approximately 1 µg of DNA is still required.

3) A totally different approach to long-read sequencing, called “virtual long read,” was initially developed by 10X Genomics (https://www.10xgenomics.com). Each long DNA fragment is encapsulated in a microfluidic droplet with an individual molecular barcode. Library preparation for short-read sequencing is then performed in each droplet. After sequencing, long reads are reconstructed by connecting the short reads according to their barcodes. This approach is known as linked-read technology. This technology had a great impact on the analysis of haplotype phasing and large genomic rearrangements [21], [22], [23]. However, 10X Genomics has discontinued the production of the linked-read sequencing method. In turn, MGI (https://en.mgi-tech.com/) introduced a different linked-read method, known as single-tube long fragment read (stLFR) [24]. In this method, a hybridization sequence that is captured by barcoded beads is inserted into DNA using Tn5 transposase, to barcode each long DNA molecule. TELL-seq was developed more recently. TELL-Seq is a method that is used to obtain information on long-range regions using next-generation short-read sequencers [25]. Compared with the PacBio and ONT sequencers, the base accuracy is higher and input DNA amount required is lower, as little as in the nanogram order, in this approach. However, a careful bioinformatics analysis is still needed, depending on the target genomes, for giving a precise interpretation of the obtained fragmented information.

The application of long-read sequencing is not limited to the analysis of human disease. For various organisms as well, these long-read technologies have collectively enabled the analysis of large genomic regions at the chromosomal level and, thus, have accelerated the (re-)construction of the reference genomes. This approach has also triggered the development of a series of novel genome assembly methods. For example, plant genomes, including those of yellow sarson, broccoli, banana, *Brassica napus*, and *Macadamia jansenii*, have been assembled using PromethION data [26], [27], [28]. Newly developed assemblers, such as Redbean [29], Flye [30], and Canu [31], were used for the analysis.

These attempts have provided a fruitful feedback to further accerarate the assembly analysis of the human genome [16], [19], [20], [32], [33]. Karen et al. tried to construct a complete telomere-to-telomere human reference genome using the MinION and CCS technologies, and partially succeeded in completely reconstructing human chromosome X [33]. For human genome analyses such as these ones, many genome assembly tools have been developed for use with long-read technologies [19], [29], [31], [34], [35]. The Shasta toolkit enabled the *de novo* assembly of human genomes with an efficient use of computational resources [19]. DipAsm enables haplotype-aware assembly using a combination of haplotype phasing and genome assembly [35]. The haplotype phasing can distinguish alleles based on SNP information. Long-read sequencing allows the determination of complete sequences and structures of genomes, including highly repetitive and variable regions, which could not previously have been identified using short-read technologies. We can now utilize more precise, albeit still incomplete, reference genomes to resolve new potential features of cancer genome aberrations, including complicated SVs located in variable and/or repetitive regions, and their haplotype-level combinations. Moreover, it is being gradually revealed that those regions are particularly polymorphic among different individuals. Therefore, in bioinformatics, projects are being started and are being coordinated toward the direction of a novel concept of “graph genome,” in which the human genome and those of other organisms cannot be and should not be analyzed assuming its linearity.

Several articles have been published that reviewed long-read sequencing technologies [1], [36], [37], [38], [39], [40], [41]. However, the technologies used for long-read sequencing are rapidly changing. Relatively few review papers in the literature have addressed the application of long-read sequencing to cancer research. In this review paper, and particularly for cancer genome biologists, we first focus on the advantages of long-read sequencing analysis (sections 1 and 2). We exemplify the recent identification and characterization of SVs in human cancer genomes (Table 1). We also describe their association with the genetic and epigenetic backgrounds at a haplotype level. For researchers of technical development, we further summarize some limitations of the recent long-read sequencing projects, namely, 1) the huge amount of input DNA required, 2) error-prone sequencing outputs, 3) presence of several genomic aberrations in cancer genomes that are too large to allow cover, even by long reads, 4) challenges in visualizing complicated genome structures, and 5) bias from reference-dependent SV detection. In this review, we describe how even very complicated cancer genome structures can be precisely identified and characterized using long-read sequencing technologies. However, for the readers with a broader background, we would like to draw attention to the later sections, in which more general features are discussed. In those sections (starting at section 3), we attempt to convey the message that a similar approach may be taken for other diseases and other organisms, also by developing new analytical tools separately.

**Table 1:** Recent research on structural variants (SVs) in cancer genomes using long-read sequencing.

Category	Sequencing technology	Cancer	Focus/findings	Reference	Published year
Structural Variant	ONT	Pancreatic cancer	Construction of an SV detection workflow for the *CDKN2A* and *SMAD4* genes	42	2016
	ONT	Brain tumors	Construction of 1-day diagnostic workflow by precise SV breakpoint detection	43	2017
	Linked read	Gastric cancer	Identification of complex *FGFR2*-related rearrangements	49	2017
	Linked read	Prostate cancer	Identification of recurrent tandem duplication of *AR* enhancers caused by *CDK12* inactivation	50	2018
	PacBio	Breast cancer	Comprehensive SV characterization of cancer cell lines, including complicated amplification of the *ERBB2* gene	51	2018
	ONT	Lung cancer	Characterization of full-length transcript sequences including fusion genes	60	2019
	ONT	Lung cancer	Identification and characterization of complex SVs (CLCLs), which aberrantly affected the gene and protein expression of cancer-related genes	61	2020
	ONT, PacBio, Linked read	Breast cancer	Precise detection and karyotype-graph integration of SV/CNVs, especially in COSMIC census genes	55	2020
	Linked read	Breast cancer	Improvement of SV detection using integrative analysis of multiple platforms	57	2020
	ONT	Ovarian cancer, Prostate cancer	Development of an analytical pipeline to detect somatic SVs from circulating tumor DNA	58	2021
	ONT	Liver cancer	Development of an analytical pipeline and construction of a catalog of somatic SVs using samples of ICGC	59	2021
Transposable elements	ONT	Melanoma, Lung cancer, Breast cancer	Development of a bioinformatics tool, “nanomonsv,” for the detection of SVs and transposable elements	63	2020 (preprint)
Methylation	ONT	Breast cancer	Development of a pipeline to detect DNA methylation named “nanoEM” using a base-conversion method and long-read sequencing	78	2021
Phasing	Linked read	Lung cancer, Colorectal cancer	A pilot study of SV and phasing analysis using the linked-read technology	21	2016
	Linked read	Colorectal cancer	Characterization of chromosomal-scale aberrations and aneuploidy with phase information	22	2017
	ONT, Linked read	Lung cancer	Identification of functional mutation candidates in regulatory regions by analyzing the transcriptional regulation and gene expression patterns of mutant alleles	23	2018
	PacBio	Lung cancer	Characterization of non-coding regions potentially associated with *EGFR* exon 19 deletion	85	2020
	Linked read	Lymphoblastic leukemia	Detection and phasing of SVs, including the *ERG* deletion and the *DUX4–IGH* fusion	89	2020

## 2 Studies of SV in human cancer genomes using long-read sequencing

There are two broad categories of computational methods for detecting SVs from long-read data: mapping-based methods and *de novo* assembly-based methods. For mapping-based SV detection, the sequence data are initially mapped to a reference genome. Long reads representing SVs should appear as a read producing a “split alignment.” For such a read, the sequence should be “split” by two or more sub-reads. These sub-reads are further mapped to a different region of the reference genome (Fig. 1A), to collectively represent an SV spanning multiple regions of the genome. For *de novo* assembly-based SV detection, a genome assembly is first constructed from long-read sequencing data. Then, differences from the reference genome are detected and extracted as SVs. The alignment-based method is more effective in detecting SVs in terms of computational cost when the reference genome exits. In addition, the method is not as affected by heterozygosity and tumor purity because only one sequence read can indicate the SV. Conversely, the de novo assembly-based method is more effective when the reference genome does not exist. In the current algorithms, the assemblers construct haploid genome regardless of heterozygosity, or assume a diploid genome. This is because the *de novo* assembly-based method cannot construct precise contigs, given the complex structure and heterozygosity of cancer genomes. Therefore, for human cancer samples, the alignment-based method is generally used.

In a pioneering study of the application of SVs for analyzing human cancers using long-read technologies, in 2016 Norris et al. used MinION sequencing of PCR amplicons from pancreatic cancer cell lines [42]. They attempted to test the ability of data generated by a MinION sequencer to detect SVs by focusing on well-characterized SVs in the *CDKN2A* and *SMAD4* genes, which are tumor suppressor genes. The authors were able to detect SVs including translocations, inversions, deletions, and the combination of inversions and translocations, which led to functional loss of the genes by reads with around 500 bp. In 2017, Euskirchen et al. attempted to develop a method to diagnose central nervous system (CNS) tumors to meet the WHO 2016 classification using MinION technology focusing on its portability. [43]. For example, codeletion of chromosome 1p-arm and chromosome 19 q-arm of the CNS tumor is one of the diagnostically relevant alterations [44], [45], [46], [47], [48]. They successfully constructed a 1-day workflow for the diagnosis of the CNS tumors, and the codeletion could be recapitulated. However, the accurate breakpoints of the codeletion remain unknown because the breakpoints probably exist in centromeric regions, which comprise highly repetitive sequences and represent ambiguous bases in the current human reference genome. They also detected the amplification of cancer-related genes, such as *EGFR*, *PDGFRA*, and *CDK4*. Greer et al. performed the linked-read whole-genome sequencing of a primary gastric tumor and two metastases from the same individual [49]. They focused on the *FGFR2* gene, in which rearrangements occur only in metastases. They also identified a complex tandem duplication with unique breakpoints in each of the metastases. These results suggest that *FGFR2*-related rearrangements have metastatic potential in gastric cancer. In 2018, Viswanathan et al. performed a linked-read whole-genome sequencing of 23 metastatic castration-resistant prostate cancers using biopsy specimens [50]. They identified a highly recurrent tandem duplication of the *AR* gene and an upstream enhancer of the *AR* gene in the context of a genome-wide tandem duplication phenotype that was introduced by *CDK12* inactivation. The amplifications were specific to the metastatic tumors. These results suggest that metastasis is related with the tandem duplication of the *AR* gene and its enhancer. Their findngs also indicated that, even in non-coding loci, SVs in a cancer genome may have an important function in tumorigenesis and resistance to treatment. Nattestad and colleagues characterized SVs in the breast cancer cell line SK-BR-3 using PacBio sequencing [51]. In their pipeline, they used the NGMLR software for mapping sequencing reads to the human reference genome, and the Sniffles software to detect SVs [52]. For the benchmarking of long reads to detect SVs, they compared the SVs identified in short-read data with those of long-read data from the same material using RT-PCR. Regarding the ability to detect SVs, the long reads were superior to the short reads. They also focused on the *ERBB2* gene, which is one of the most important genes for tumorigenesis and diagnosis in breast cancer and amplified in the SK-BR-3 cells. They identified a complex structure of SVs associated with this particular gene, including nested duplications and five translocations. The diagnosis of amplification of the *ERBB2* gene is generally conducted by fluorescence *in situ* hybridization. There are some targeted drugs for the *ERBB2* amplification, for example, trastuzumab [53]. However, these drugs were not effective in some patients with *ERBB2*-amplified breast cancer [54]. Therefore, the elucidation of the genomic structure of the amplification is important.

For methodological developments aimed at detecting SVs more precisely, combination analyses of long-read sequencing and short-read sequencing have also been conducted in several cancer genomes. Aganezov et al., who work in the same research group as Nattestad and colleagues, performed deep, whole-genome sequencing of a breast cancer cell line and two breast cancer clinical samples using ONT PromethION, PacBio, 10X linked-read sequencing, and Illumina sequencing, to detect and characterize SVs precisely [55]. The authors characterized allele-specific SVs by reconstructing haplotype-specific cancer karyotype graphs [56] in cancer-related COSMIC census genes. Sethi et al. characterized SVs from MCF-7, a breast cancer cell line, and from a primary breast cancer tumor using Illumina short-read sequencing, with a 10X linked-read sequencing being used for benchmarking [57]. Those authors showed that the combination of linked-read sequencing and Illumina sequencing increased the specificity and sensitivity of the detection of SVs. These studies collectively suggest that integrative studies using both short-read and long-read sequencing should be useful for the robust detection of SVs. However, it is costly in terms of sequencing and computational costs to employ both of these approaches in every case.

Among the most recent studies of SVs in cancer, in 2021, Valle-Inclan et al. developed a method to detect SVs from circulating tumor DNA at a low sequence depth (for example, 2–4×) using ONT long-read sequencers [58]. This method aims to track tumor burden using somatic SVs as biomarkers from liquid biopsies and was indicated the usefulness of the method. Fujimoto et al. tried to construct a catalog of polymorphic and somatic SVs from long-read sequencing data based on ONT MinION sequencing of 11 Japanese liver cancers that had been previously sequenced by the International Cancer Genome Consortium [59]. For this purpose, they developed a new analytical pipeline called CAMPHOR. Subsequently, they attempted to identify the mechanism underlying the generation of the called SVs.

In our own recent study, we characterized the transcripts of fusion genes in lung adenocarcinoma cell lines using MinION full-length cDNA sequencing [60]. The junction of a fusion gene of *CCDC6* and *RET*, which is a driver gene of the LC2/ad cell line, could be identified with precision. We also performed whole-genome sequencing of five non-small cell lung cancer cell lines and 20 lung cancer clinical samples using MinION and PromethION. We identified complex structural aberrations, named Cancerous Local Copy-number Lesions (CLCLs) [61]. CLCLs are complicated SVs combining local tandem duplication, inversion, and/or micro deletions. CLCLs were found in tumor suppressor genes, such as *STK11*, *NF1*, and *PTEN* in the RERF-LC-KJ, RERF-LC-MS, and PC-14 cell lines, respectively (Fig. 1B). We also demonstrated that the presence of CLCLs led to aberrant transcription of RNA and affected the function of the proteins produced by the genes involved in them. The driver genes in the two cell lines remain unknown. Therefore, these results may provide new insights regarding the driver events of cancer initiation and progression. Furthermore, we detected CLCL candidates in clinical samples, which indicated that CLCL events can occur not only in cell lines, but also in real clinical samples. We are convinced that several very complicated SVs, such as CLCLs, play important roles in tumorigenesis and/or cancer progression, and that these SVs need to be precisely identified using long-read sequencing technologies.

## 3 Transposable elements and SVs

LINE-1 retrotransposition can produce rearrangements in genes that are functionally important in cancer. The PCAWG project explored LINE-1 insertions in 2954 cancer genomes from 38 histological cancer subtypes from the International Cancer Genome Consortium and The Cancer Genome Atlas using Trafic-mem [12]. They performed short-read sequencing, and the data obtained revealed that LINE-1 insertions caused somatic SVs in the genomes of patients with cancer. However, the size of LINE-1 insertions is, at most, 6 kb [62], and it is hard to resolve the complete inserted sequences and to identify accurate inserted positions based on short reads.

LINE-1 insertions and LINE-1 transposition-driven SVs should be more accurately and easily detected using long-read, rather than short-read, sequencing data in terms of sequence read length. However, long reads are error-prone regarding base accuracy; thus, as improved method needs to be developed to detect transposable elements precisely, rather than applying the current methods to detect SVs. Shiraishi et al. developed a tool named “nanomonsv” to detect SVs and mobile element insertions from tumor and matched non-cancer long-read sequencing data [63]. In nanomonsv, putative SVs and supporting reads are detected based on sequencing reads mapped to the reference genome. Consensus sequences are generated based on the clustered supporting reads, and SV breakpoints are identified by a one-time jump Smith–Waterman algorithm. Finally, putative SVs are confirmed by remapping the SV sequence to the reference genome and comparing it with matched control data. Using this pipeline, the researchers characterized LINE-1 insertions in cancer cell lines. In another preprint article, Pascarella et al. used high-throughput target-capturing short-read sequencing data (capture-seq data) and ONT MinION long-read sequencing of retroelements to show that non-allelic homologous recombination of Alu and LINE-1 in human genomes leads to the presence of recombination hotspots in SVs [64]. They also developed a new bioinformatics pipeline, named TE-reX. This program supports capture-seq and long-read sequencing data using an alignment algorithm termed LAST [65]. These researchers discuss the potential biological relevance of these retroelements in the genomes of patients with cancer, as well as in the genomes of individuals with Parkinson’s disease and Alzheimer’s disease.

Short-read sequencing is unable to decipher the complete sequences of long-range insertions. Unfortunately, the detection and characterization of transposable element insertions using long-read sequencing are still at the developing stage, and robust bioinformatics methods have not been constructed. However, many research groups are focusing on transposable elements, as described above. With the rapid development and spread of long-read analyses, research projects on retrotransposons progress in the near future.

## 4 DNA methylation and SVs

**Fig. 2:**
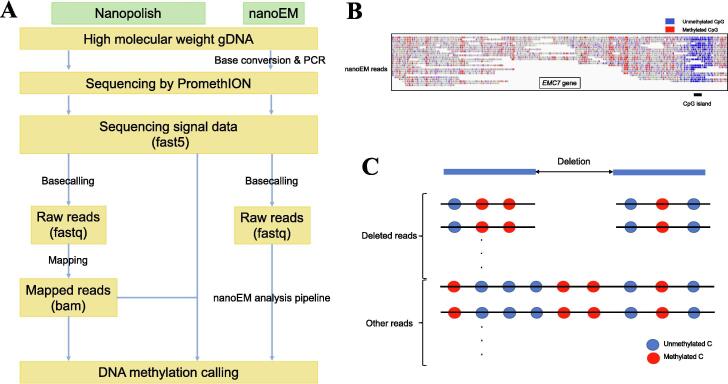
SV calling and DNA methylation detection. (A) Workflow of DNA methylation detection using Nanopolish and nanoEM using ONT PromethION. Extraction of high molecular weight genome DNA (gDNA) is common between the Nanopolish and nanoEM methods. For the Nanopolish method, after whole-genome sequencing using an ONT sequencer, a fast5 format signal file from ONT sequencing and the bam format file produced from mapping sequence reads to reference genome, are needed as input. For nanoEM, before sequencing, base conversion from unmethylated cytosines to uracils and PCR are needed. After sequencing, the nanoEM analysis pipeline developed by us is conducted. (B) An example of nanoEM. The DNA methylation status of nanoEM long reads was shown in IGV. Blue: unmethylated CpG, red: methylated CpG. The IGV indicates that CpGs in the promoter of the *EMC7* gene were unmethylated, which suggest that the gene can be transcriptionally active. (C) Example of the simultaneous visualization of a deletion and DNA methylation status of each sequence read. Reads supporting the deletion represent a different methylation status vs. normal reads). (For interpretation of the references to colour in this figure legend, the reader is referred to the web version of this article.)

It has been suggested that aberrant DNA methylation in the genome may contribute to cancer development [66]. Both the ONT and PacBio long-read technologies can detect the 5-methylcytosines (5 mCs) of CpG dinucleotides directly using signals from a sequencing electogram. This detection is based on the distinction between signals from a methylated and an unmethylated cytosine. The approaches are as follows (Fig. 2A).

1) For the Nanopore sequencer, several tools were developed for methylation calling [67], [68], [69], and several benchmarking results have been published [70], [71], [72]. For example, Nanopolish, which is pioneering and one of the most applicable to cancer samples, was developed by using a hidden Markov model to detect 5 mCs in CpG sites according to the differences in signals [67]. ONT also developed a methylation-calling tool, Megalodon (https://github.com/nanoporetech/megalodon). This tool compares sequence scores between the methylated sequence and the reference sequence using a reference-anchored base-calling output via a neural network method. Lee et al. combined Nanopore sequencing data and NOME-seq data, in which DNA accessibility was detected using GpC methyltransferase, and designated this method nanoNoMe [73], [74]. This method also uses Nanopolish to detect CpG methylation on the Nanopore side.

2) For the PacBio sequencer, Tse et al. developed a method to detect 5 mCs using SMRT sequencers [75]. This algorithm is based on the difference in inter-pulse duration and pulse width between methylated and unmethylated cytosine. These researchers constructed a convolutional neural network model using the sequencing data from the methyltransferase-treated DNA and the unmethylated DNA.

3) Methods combining long-read sequencing and base conversion have also been developed. Liu et al. established long-read Tet-assisted pyrimidine borane sequencing (lrTAPS) for targeted sequencing [76]. In the pipeline of lrTAPS, methylated cytosine to uracil base conversion is performed using hTet2 and pyrimidine borane, and long-read sequencing is conducted after PCR.

Our group has also developed a method combining Nanopore-type sequencing and Enzymatic Methyl-seq (EM-seq) [77], named nanoEM [78]. EM-seq converts bases from unmethylated cytosines to uracils using an enzymatic reaction that prevents the fragmentation of DNA libraries during bisulfite sequencing, which is used to determine the pattern of DNA methylation. We applied nanoEM to two breast cancer cell lines and three breast cancer clinical samples, to characterize their methylation status, using a newly developed bioinformatics pipeline. We obtained sequencing results of about 5 kb at N50 length. We evaluated the nanoEM by comparison with the results of whole-genome bisulfite sequencing, EM-seq with Illumina sequencing, and Nanopolish using long-read whole-genome sequencing. We detected unmethylated CpG islands in the promoter regions of the *ERBB2* and *PGR* genes, which are used in the classification of breast cancer subtypes. We also detected differentially methylated regions in cancer-related genes, such as *CMYA5*, *TSLP*, *ZNF503*, and *ZNF217*, which suggest that the methylation status of these genes may be involved in tumorigenesis or cancer progression. In addition, we found that nanoEM could analyze SVs and the methylation status of their surrounding regions simultaneously (Fig. 2B). Several studies reported by other groups have indicated that LINE-1 transposition, for example, is associated with DNA methylation status [79], [80]. These studies have indicated that long-read direct methylation sequencing is also capable of detecting allele-specific methylation.

In cancer, DNA methylation plays an important role in tumorigenesis or cancer progression [81]. For example, CpG islands located in the promoter of tumor suppressor genes can be methylated, leading to transcriptional inhibition of the tumor suppressor genes. Methylation can be detected by bisulfite sequencing using short-read sequencers. Short-read sequencing can detect DNA methylation at a specific base more accurately than does long-read sequencing. Conversely, long-read sequencing can detect DNA methylation in a wider range than does short-read sequencing. This is because integrative analyses can be conducted, for example, DNA methylation, SVs, and haplotype information. It was reported that the accuracy of methylation calling from the long-read sequencing had high concordance with the short-read sequencing [78]. Therefore, DNA methylation analyses using long-read sequencing will flourish.

## 5 Haplotype phasing and SVs

Haplotype phasing constructs the SNP sequence of each allele, which can distinguish the SNP patterns of maternal and paternal alleles in a human genome. In a genome responsible for cancer, haplotype phasing involves distinguishing the chromosomal background of the alleles in which aberrant events, such as somatic point mutations and SVs, occur. In 2016, Zheng et al. performed linked-read sequencing of HapMap trio samples (NA12878, NA12877, and NA12882), the lung cancer cell line NCI-H2228, and primary colorectal adenocarcinoma [21]. They aimed to assess the phasing performance using well-annotated HapMap samples. Subsequently, the authors identified an *EML4–ALK* fusion in NCI-H2228 cells. This mutation is known as a driver mutation of lung adenocarcinoma using exome-based phasing [82], [83]. In 2017, Bell et al. performed linked-read sequencing of tumor and dysplasia samples from three primary patients with colorectal adenocarcinoma, matched normal samples, a metastatic sample, a colorectal cancer cell line, and a cell line with trisomy of chromosomes two and 21 [22]. They developed an analytical pipeline to detect large chromosomal changes and aneuploidy using normalized barcode counts. This pipeline was used to detect a significant difference between the tumor and dysplasia samples and the matched healthy samples with respect to the distributions of the normalized barcode counts of each haplotype. After validating the method using the trisomy cell line data, they applied the method to clinical samples, and successfully identified an allelic imbalance derived from the SVs and aneuploidy in a colorectal cancer genome. In 2018, Sereewattanawoot et al., in our work group, reported the haplotype phasing of 23 lung adenocarcinoma cell lines using linked-read sequencing [23]. They validated the results of the phasing using ONT MinION sequencing, and attempted to identify an association between the regulatory mutations and their transcriptional consequences using haplotype phasing and previously generated multi-omics information of whole-genome, transcriptome, and epigenome sequencing data, including DNA methylation and eight histone modifications [84]. An SNV located in the regulatory region of the *NFATC1* gene in the RERC-LC-Ad1 lung cancer cell line and allele-specific transcription with the mutation was detected. In 2020, Cook et al. focused on two deletions in exon 19 of the *EGFR* gene [85], which is the most important driver gene of lung adenocarcinoma, being responsible for the disease in 50% of Japanese patients with lung adenocarcinoma [86]. The authors conducted PacBio CCS sequencing of the samples from two patients with lung adenocarcinoma, and conducted haplotype phasing using WhatsHap [87]. They tried to unveil the mechanism of the *EGFR* exon 19 deletion by searching a non-coding region potentially associated with the deletion using a previously published Alu-element-based instability model [88]. This model considers two adjacent Alu elements with opposite orientations, which can align to form a DNA loop structure. This structure can lead to a double-strand break, causing a large deletion. Nordlund et al. conducted linked-read whole-genome sequencing of 12 acute lymphoblastic leukemia samples, to detect and phase SVs [89]. They evaluated the ability of linked-read sequencing to detect and phase SVs from biobanked DNA, even at 10 × coverage. They identified a previously known heterozygous deletion of the *ERG* gene in a patient carrying the *DUX4–IGH* fusion gene, using haplotype information.

Although haplotype phasing using long-read sequencing is directly linked to the SNPs on a read, using short-read sequencing consists simply in the imputation of alleles using statistical methods. This is because haplotype phasing using long-read sequencing can analyze SVs simultaneously. However, SNP calling is a drawback of long-read sequencing, as it has a high sequencing-error rate. Therefore, we should consider a combinatorial analysis of short-read and long-read sequencing data to perform haplotype phasing.

## 6 Summary and outlook

Long-read sequencing technologies have produced significant advances in the elucidation of aberrant genome structures, including key disruption events that are important in cancer-related genes. However, it remains difficult to apply these technologies to the diagnosis of clinical cancer specimens on a day-to-day basis. For example, long-read sequencing technologies, including ONT and PacBio sequencers, generally require microgram-order DNA for library preparation for whole-genome sequencing. The amounts of DNA collected as clinical samples are occasionally very small, and the fraction that can be used for the molecular diagnosis is limited. This is a significant technical burden for the clinical application of long-read sequencing for cancer diagnosis. For the wider application of long-read sequencing, the development of technologies that require smaller amounts of starting materials should be a priority.

Error-prone long-read sequencing instruments (with the exception of PacBio CCS technology, with over 99% accuracy) should facilitate deep sequencing to detect variants. To distinguish between sequencing errors and true variants, it has been estimated that at least 8 × coverage is needed [90]. Clinical tumor samples are usually mixed with normal cells, a situation that requires even deeper coverage. For clinical applications, it is not realistic to perform multiple deep sequencing using multiple sequencing technologies, and to obtain consensus results of SV calling, because of the limited amount of sample available. To obtain sufficient sequencing depths of long-read data, target enrichment by hybridization capture or CRISPR-based methods could be used. Whole-genome amplification may also be useful, despite the limited length (around 5 kb with nanoEM, for example) [78]. Very recently, ONT has announced early access to the PromethION flowcell R10.3 version, which has much higher sequencing accuracy than previous versions of flowcell. This new platform should achieve the sequencing quality of 99% (Q20) at the single read, which may solve the low base accuracy of the current long-read sequencing and lead to improvement of downstream analyses, such as transposon insertion detection, methylation analysis, haplotype phasing, and *de novo* assembly.

**Fig. 3:**
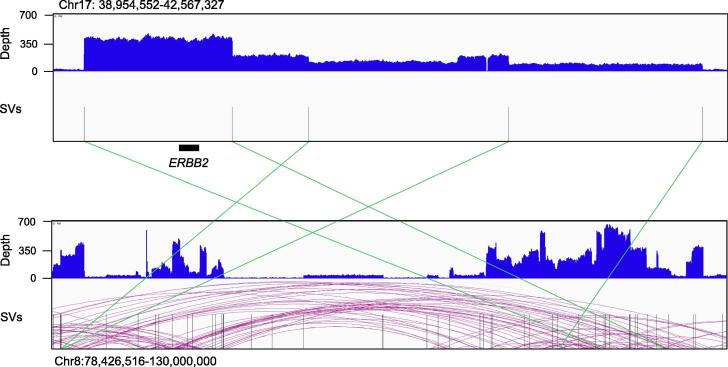
*ERBB2* amplification of the SK-BR-3 cell line. Amplification of a region including the *ERBB2* gene in chromosome 17 with four translocations to chromosome eight detected using ONT PromethION whole-genome sequencing data at 46× depth [51] (upper panel). The upper-half y-axis indicates the sequencing depth. The amplification spans 3.6 Mb. The size of the amplification is larger than any sequence reads, so the structure of the amplification cannot be resolved precisely. The green lines indicate the breakpoints of translocations to chromosome eight (lower panel), as called by Sniffles. The *ERBB2* gene is very important for breast cancer in terms of diagnosis and treatment. The reconstruction of the *ERBB2*-related SVs may lead to a detailed effect of existing *ERBB2*-targeting drugs. (For interpretation of the references to colour in this figure legend, the reader is referred to the web version of this article.)

From a wider perspective, the structures of SVs at a level over 1 Mb cannot be resolved using current long-read sequencers, although this situation may improve in the near future. For example, amplification including the *ERBB2* gene in the SK-BR3 breast cancer cell line spanned a 3 Mb region with multiple translocations on chromosome eight (Fig. 3) [51], [61]. The longest read length of the long-read sequencers is at most a few megabases, and the N50 length of sequencing is at most around 50 kb. It remains difficult to conduct the assembly of cancer genomes, because of heterogeneity and heterozygosity. New algorithms for investigating these phenomena should also be developed.

The visualization of SVs is also an unsolved problem. Established genome browsers, such as IGV and RIBBON, present the genome as a linear structure [91], [92], [93]. SVs widely distributed in genomes cannot be visualized in one window. This is stressful for end-users, who must visually inspect the candidate SVs. To solve this problem, Yokoyama et al. developed the MOdular Multi-scale Integrated Genome graph browser (MoMI-G), a genome browser based on a genome graph. MoMI-G can visualize SVs in one window, although the robustness remains insufficient for manipulation of the browser.

Lastly, but no less importantly, we need to manage reference biases in mapping-based SV detection. A genome graph structure can solve this problem. As this remains a pioneering field, no agreed-upon format for a genome graph has been established [94]. The human reference genome is being intensively reviewed. The telomere-to-telomere project has produced a human genome assembly from 5′ telomere to 3′ telomere without any gaps, using long-read sequencing [33], although the haploid cell line CHM13 was used for this project, implying that this genome assembly should be carefully considered with non-diploid assembly. Through the continuous efforts of many researchers in many projects, we believe that long-read sequencing will produce a human reference genome in which difficult loci, such as repetitive regions, and variations among diverse populations still exist, to yield a more complete structure in the near future. Such a reference genome would enable progression to the precise identification and characterization of cancer SVs, which would bring new insights into cancer genomics at the biological and clinical levels.

## CRediT authorship contribution statement

**Yoshitaka Sakamoto:** Writing - original draft, Visualization, Writing - review & editing. **Suzuko Zaha:** Visualization. **Yutaka Suzuki:** Writing - review & editing. **Masahide Seki:** Writing - review & editing. **Ayako Suzuki:** Writing - review & editing, Supervision.

## Declaration of Competing Interest

The authors declare that they have no known competing financial interests or personal relationships that could have appeared to influence the work reported in this paper.
